# Heterogeneity in mechanisms of emergent resistance in pediatric T-cell acute lymphoblastic leukemia

**DOI:** 10.18632/oncotarget.11233

**Published:** 2016-08-11

**Authors:** Babasaheb D. Yadav, Amy L. Samuels, Julia E. Wells, Rosemary Sutton, Nicola C. Venn, Katerina Bendak, Denise Anderson, Glenn M. Marshall, Catherine H. Cole, Alex H. Beesley, Ursula R. Kees, Richard B. Lock

**Affiliations:** ^1^ Leukaemia Biology Program, Children's Cancer Institute, Lowy Cancer Research Centre, University of New South Wales, Sydney, New South Wales, Australia; ^2^ Division of Children's Leukaemia and Cancer Research, Telethon Kids Institute, University of Western Australia, Perth, Western Australia, Australia; ^3^ Molecular Diagnostics, Children's Cancer Institute, Lowy Cancer Research Centre, University of New South Wales, Sydney, New South Wales, Australia; ^4^ Division of Bioinformatics and Biostatistics, Telethon Kids Institute, University of Western Australia, Perth, Western Australia, Australia; ^5^ Kids Cancer Centre, Sydney Children's Hospital, Sydney, New South Wales, Australia; ^6^ School of Paediatrics and Child Health, University of Western Australia, Perth, Western Australia, Australia

**Keywords:** acute lymphoblastic leukemia, relapse, drug resistance, xenograft, pre-clinical testing

## Abstract

Relapse in pediatric T-cell acute lymphoblastic leukemia (T-ALL) remains a significant clinical problem and is thought to be associated with clonal selection during treatment. In this study we used an established pre-clinical model of induction therapy to increase our understanding of the effect of engraftment and chemotherapy on clonal selection and acquisition of drug resistance *in vivo*. Immune-deficient mice were engrafted with patient diagnostic specimens and exposed to a repeated combination therapy consisting of vincristine, dexamethasone, L-asparaginase and daunorubicin. Any re-emergence of disease following therapy was shown to be associated with resistance to dexamethasone, no resistance was observed to the other three drugs. Immunoglobulin/T-cell receptor gene rearrangements closely matched those in respective diagnosis and relapse patient specimens, highlighting that these clonal markers do not fully reflect the biological changes associated with drug resistance. Gene expression profiling revealed the significant underlying heterogeneity of dexamethasone-resistant xenografts. Alterations were observed in a large number of biological pathways, yet no dominant signature was common to all lines. These findings indicate that the biological changes associated with T-ALL relapse and resistance are stochastic and highly individual, and underline the importance of using sophisticated molecular techniques or single cell analyses in developing personalized approaches to therapy.

## INTRODUCTION

Pediatric T-cell acute lymphoblastic leukemia (T-ALL) is a highly aggressive cancer, with clonal heterogeneity contributing to the progression of disease and resistance to treatment. Patients often show dismal prognosis after first relapse, with only 0% to 25% of patients achieving durable remission after second-line treatment [1-7]. The majority of T-ALL relapses occur during therapy or soon after completion of therapy, with resistance to glucocorticoids considered to be one of the main contributors to therapeutic failure [8-10]. Development of primary resistance can be considered either in terms of clonal evolution or the selection of a resistant sub-clone that was already present at low numbers at the time of diagnosis [11-15], resulting in the outgrowth of aggressive and resistant disease with adverse clinical outcome.

Patient-derived xenograft (PDX) models have been widely utilized to gain insight into clonal evolution and mechanisms of resistance to chemotherapy in ALL [16-20]. However, to date no molecular studies of T-ALL clonal selection and/or evolution in response to standard remission-induction therapy have been performed in PDX models. In 2014 our group established a pre-clinical mouse model of T-ALL relapse [21]. A unique aspect of this model is that it uses a four-drug combination therapy with a repeated-block design to model, as closely as possible, current ALL induction-therapy protocols. In the present study we have used this model to examine in greater detail the process of drug-resistance acquisition and clonal evolution *in vivo*. Our findings indicate that mechanisms of resistance to remission-induction are highly heterogeneous, supporting the continued development of individualized approaches to leukemia therapy.

## RESULTS

### Kinetics of engraftment and relapse in a pre-clinical model of T-ALL therapy

To examine the role of engraftment and chemotherapy in the selection of leukemic cells, a previously optimized, clinically relevant four drug model of induction therapy was used in immunocompromised (NSG) mice engrafted with diagnostic bone marrow samples from T-ALL patients [21]. In that earlier study, two xenograft lines (ALL-27 and ALL-31) were established that demonstrated acquired drug resistance in response to treatment [21]. In the present study six additional xenograft lines were established and treated in the same way, two at second passage (ALL-42 and ALL-44) and four at first passage (ALL-46, ALL-47, ALL-72 and ALL-73), in regards to the stage of their serial engraftment in mice. In total, xenografts derived from the diagnosis specimens of eight T-ALL patients are presented (Table 1), consisting of one with refractory disease (ALL-31), one with chemo-resistant disease who died early (ALL-44), three with early relapse (< 18 months) in the bone marrow (ALL-42, -44, -46, -73), two with early isolated central nervous system relapse (ALL-27, -47) and one still in first remission (ALL-72). Following engraftment, all xenografts were treated for four weeks with VCR, DEX, ASP, and DNR (the ‘VXLD' module, Supplementary Table S1A). Disease onset was monitored in peripheral blood by determining the percentage of human CD45+ cells and, upon disease re-occurrence, treatment was continued for a further two weeks with half doses of VCR, DEX and L-ASP (the ‘1/2 VXL' module, Supplementary Table S1A). The combination of both treatment blocks was designed to replicate therapy for newly diagnosed ALL whilst accounting for toxicity, and is referred to as the ‘VXLD2′ schedule.

**Figure 1:**
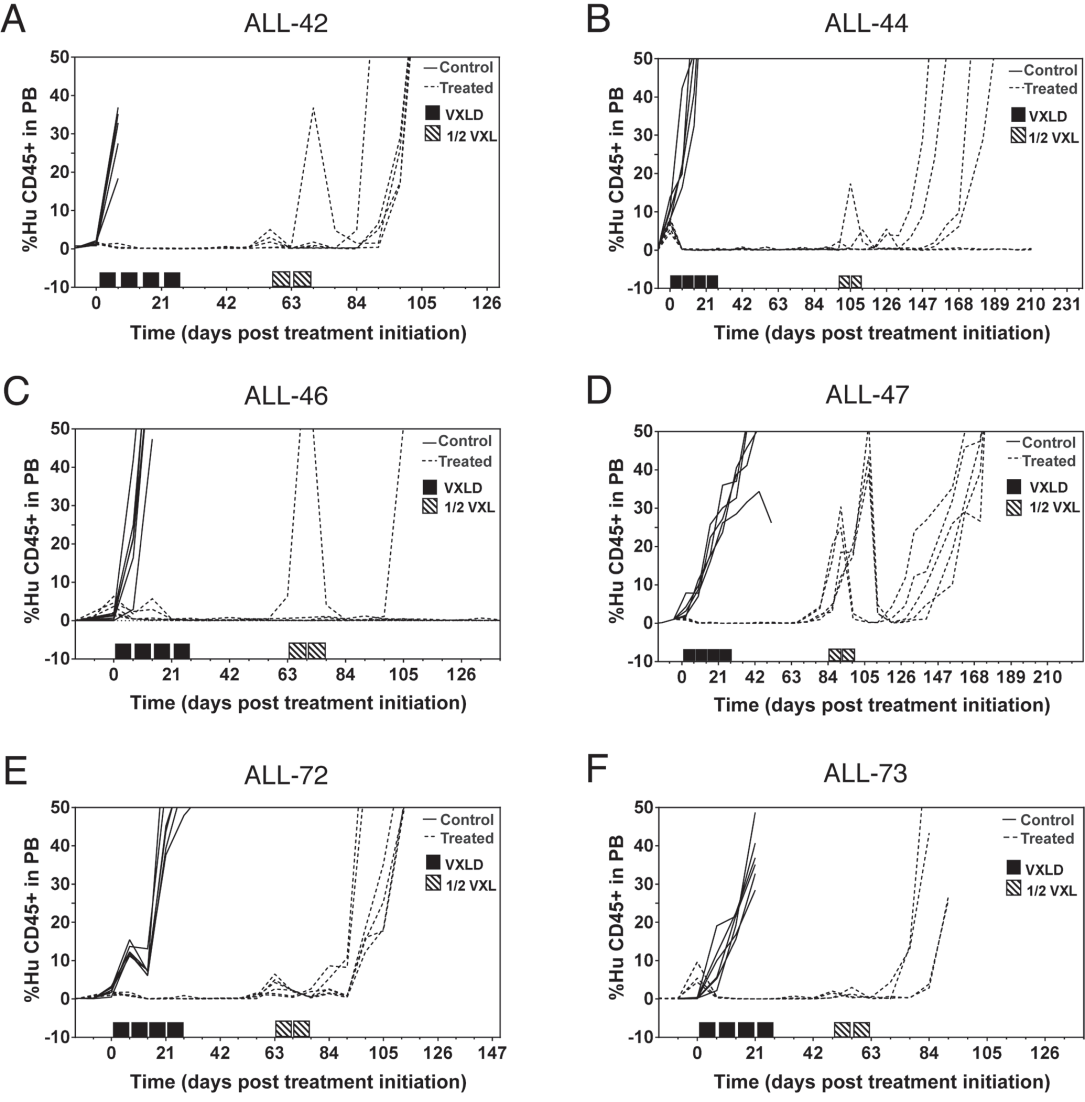
Development of drug-resistant T-ALL xenografts NSG mice were inoculated with patient biopsy samples and treated with the induction-therapy VXLD2 schedule (or saline) to create multidrug resistant sublines. The percentage of human CD45^+^ cells in the peripheral blood was used to assess leukemia progression, in xenografts treated either during second passage **A.** & **B.** or first passage **C.**-**F.** in regards to serial engraftment of the patient diagnostic specimen.

In all six of the newly derived xenografts, the initial four-week treatment module clearly delayed disease progression (Figure 1), but in each case disease re-emerged 28-77 days after the last week of treatment. However, the response of each of the lines to the second round of therapy (1/2 VXL) varied considerably, with disease reoccurring in only some of the engrafted animals (Figure 1, and Supplementary Table S1B).

To further probe the behavior of T-ALL samples in this pre-clinical model, we repeated the *in vivo* drug selection protocol to establish independent xenograft lines using diagnosis samples from two patients from whom we had generated ALL-44 and ALL-46. These xenografts are referred to as ALL-44 ‘repeat' and ALL-46 ‘repeat', and they were treated in exactly the same way as described above. We observed that in case of the ALL-46 ‘repeat', the kinetics of engraftment (Supplementary Figure S1A) exactly matched that of the original xenograft (Figure 1C). In contrast, a stark difference in the engraftment pattern was observed for ALL-44 ‘repeat', where none of the mice ‘relapsed' following the initial four-week treatment module (Supplementary Figure S1B), whilst in the original ALL-44 xenograft disease remained evident in four out of six mice even after both treatment modules (Figure 1B). The variability in the response to treatment for ALL-44 (summarized in Supplementary Table 1B) indicates that there may be a stochastic element to the selection or evolution of clones with the capacity to survive drug-therapy.

**Table 1:** Patient characteristics and risk factors for diagnostic bone-marrow samples used in T-ALL patient-derived xenografts (PDX)

T-ALL PDX (patient ID)	Clinical outcome	Age at Dx (yrs) & sec	Dx WBC (10^9^)	NCI risk group	D7 Pred response	End of Induction MRD	Length of CR1 (months)	Current clinical status	Cytogenetics	Immunophenotype
ALL-27 ^*^	CNS rel	8.5 M	526.2	HR	poor	-ve	10	Died	46, XY [24]	CD45+/HLA-DR-/CD19-/22-/10-/cyto3+/4+/ 8+/2+/7+/34-/13-
ALL-31 ^*^	Res Dis	10.1 M	212.2	HR	poor	4 × 10^-1^	0	Died	46,XY,del(6)(q21),del(ll)(q23)[4]/46,XY[14]	HLA-DR/CD19-/10-/2+/3+/5+/ 7+/TCRab+/Tdt+
ALL-42	BM rel	2.6 M	N/A	N/A	N/A	2×10^-4^	16	Died	46,XY[20]	HLA-DR/CD19/10-/2+/3+/4+/8+/5+/7+/13-/33-/34-
ALL-44	Died in CR1	3.4 M	177.4	HR	poor	9×10^-3^	13	Died	46, XY[20]	HLA-DR/2+/m3-/cyto3+/4-/8-/5+/7+
ALL-46	BM rel	15.8M	380	HR	good	<1×10^-4^	16	Died	46, Y,der(X) t(X;17)(q28;ql.2),t(8;14)(q24;qll.2)[15]/46,XY[5]	CD45+/HLA-DR-/CD19-/22-/10-/3+/5+/8+/2+/ 7+/4-
ALL-47	CNS rel	6.5 F	45.5	SR	good	<5×10^-5^	15	Alive (CR2 84m)	46, XX,del(6)(q21q25),del(9)(pl2),t(ll;14)(pl5;qll)[15]/46XX	HLA-DR-/CD19/2+/3+/5+/7+/4-/8+/34+/Tdt +
ALL-72	Event free	6.3 M	219.1	HR	N/A	N/A	64	Alive (CR1 64m)	46,XY,del(6)(q21q23),t(9;13)(p22;ql4)[10]	CD45+/HLA-DR-/CD19-/22-/10/3+/5+/4+/8+/ 2+/7+/34-/13-
ALL-73	BM rel	0.9 M	173	HR	N/A	2×10^-2^	12	Died	46,XY,t(6;7)(q23;q34)[5]/46,XY[3]	HLA-DR-/CD19-/10-/m3-/cyto3+/4+/8-/5+/2+/7+/34-/13-/33-/38+

* ALL-27 & ALL-31 were reported previously (Samuels et al 2014); cyto3+, cytoplasmic CD3 positive; Res Dis, resistant disease; CNS, central nervous system; BM, bone marrow; Dx, diagnosis; rel, relapse; DOD, died of disease; CR, clinical remission; HR, high risk; SR, standard risk; N/A, not available; D7 Pred response, Day 7 Prednisone Response (‘good or poor’, according to BFM definition); MRD, minimal residual disease; D35, Day 35; WBC, white blood cell count

### Effect of engraftment and drug-treatment on T-ALL clonal markers

To evaluate the selection pressure caused by engraftment in NSG mice, we explored the *Ig/TCR* and *SIL-TAL1* gene rearrangements of diagnostic patient samples, and their corresponding xenografts at first, second or third passage (Supplementary Data File1). Assessments were performed using a panel of 25 clonal markers for the patient samples and first two xenografts ALL-42 and ALL-44, whilst for the remaining xenografts, the major markers (2-3 gene rearrangements per line) were sequenced by Sanger sequencing and/or quantified by RQ-PCR.

Three of the six patients in the present study had bone marrow relapses (ALL-42, ALL-46, ALL-73). Comparison of their diagnosis and relapse specimens using the *Ig/TCR* markers showed that all markers detected at diagnosis were conserved in the patients' relapse samples and in all the primary and secondary passages of their xenografted cells, independent of chemotherapy treatment. Examining the diagnostic/relapse pairs there was only one change observed in either the pattern of gene rearrangements or in the nucleotide sequence of selected markers (Supplementary Data File1). The *SIL-TAL1* rearrangement was only found in ALL-42 and was consistently present in both the patient and all derived PDX cells, with no evidence of evolution. The patient corresponding to ALL-73 acquired a TRB marker at relapse that was not detected in any of the ALL-73 PDX samples. Backtracking of this marker in the patient's earlier samples using the specific RQ-PCR test revealed this marker was present at a very low level (< 0.1%) at diagnosis and responded more slowly to chemotherapy. The same test confirmed its absence in all of the derived xenograft cells.

The retention of diagnosis clonal markers at relapse is consistent with our laboratory's experience in the routine testing of T-ALL patients for MRD, as well as reports in the literature [3, 19, 22]. There was a slightly lower level of MRD at relapse for ALL-42 due to a lower percentage of blasts at relapse than diagnosis. The much lower level of blasts in patient ALL-47 (2% compared to diagnosis) is indicative of molecular BM relapse at the time of isolated CNS relapse by clinical criteria. Interestingly for ALL-46, ALL-47 and ALL-73, there were patient samples available for intermediate time points between diagnosis and relapse, from which one can clearly trace the course of the disease in these patients i.e. initial response to therapy, as evidenced by a decrease in the selected clonal markers to an undetectable level (MRD negative), followed by a subsequent increase in the marker before clinical relapse.

Upon primary engraftment of all six diagnostic samples (ALL-42, ALL-44, ALL-46, ALL-47, ALL-72 & ALL-73) into NSG mice, there were clonal markers in these first passage mice that remained stable in comparison to the diagnosis specimen, both in terms of quantification and nucleotide sequence (Supplementary Data File1). In ALL-42 and ALL44, in which all potential markers were evaluated, there was also evidence of some minor changes to the clonal composition of the leukemic compartment, something not entirely unexpected given the selection pressure likely to be exerted by placing human cells into a mouse (albeit immunocompromised) host. However, the additional bands seen by heteroduplex electrophoresis were polyclonal by Sanger sequencing.

From each xenograft line that did not show treatment resistance at first passage (all except ALL-72), we harvested cells from a single untreated control mouse with markers most resembling the respective patient diagnostic sample, and placed these into multiple recipient mice (i.e. 2^nd^ passage). In the case of ALL-46, cells were additionally engrafted from 2^nd^ passage mice into a 3^nd^ passage. For each xenograft we selected and followed one or two Ig/TCR markers through each stage, by both RQ-PCR and Sanger sequencing (Supplementary Data File1). In every case, the selected marker in control mice remained identical in both quantity and nucleotide sequence to that seen in both the respective patient and first passage mice, indicating that so far as can be determined from *Ig/TCR* gene rearrangements, the clonal composition of the leukemic compartment remains largely stable through serial engraftment in NSG mice. Similarly, treatment of the mice with multidrug therapy (either VXLD2 for the purpose of modelling induction therapy, or ½ VXL for the purpose of maintaining baseline drug-selection pressure in subsequent expansions) did not affect either the quantity or the sequence of the clonal markers being tracked (Supplementary Data File1). Together these data indicate that although there is some selection pressure at first passage, these PDX models largely preserve the *Ig/TCR* gene rearrangements observed in the original diagnosis samples, and that *in vivo* drug-treatment (just as we observe at patient relapse) does not substantially alter the pattern of major clonal markers detected by this technique.

### *Ex vivo* assessment of drug resistance in relapsing xenografts

**Figure 2:**
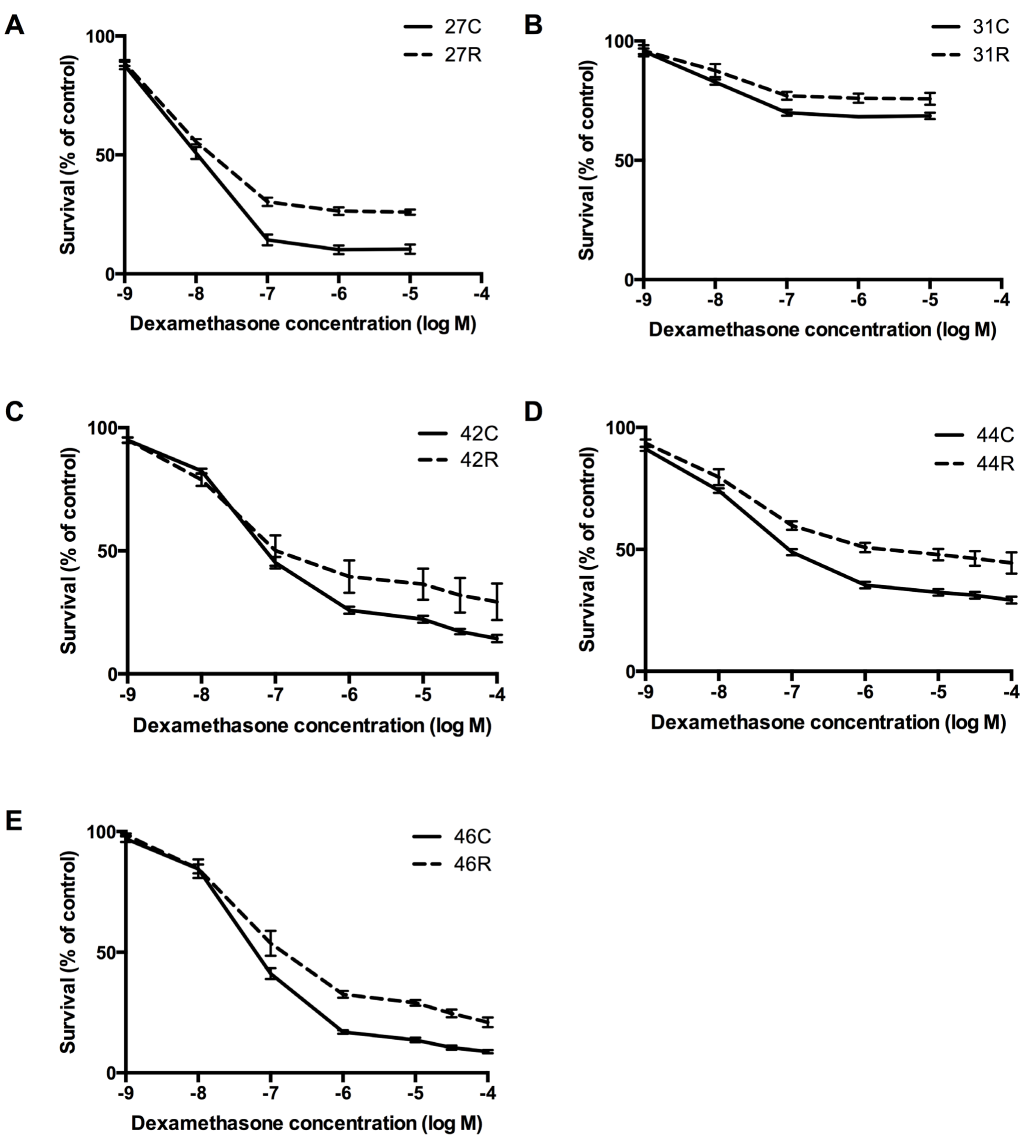
Assessment of drug resistance in relapsing xenografts Lines in which disease re-emerged (‘R') following multidrug VXLD2 induction therapy, and their passage-matched controls (‘C'), were assessed *ex vivo* for acquired drug resistance to single agent DEX: **A.** ALL-27, **B.** ALL-31, **C.** ALL-42, **D.** ALL-44 & **E.** ALL-46. The Alamar blue assay was used to measure cell survival (mean ± SEM) following a 48-hour drug exposure; each treatment arm (‘R' or ‘C') represents the average of 4-6 independent mice, with three technical replicates performed for each mouse.

We next wished to determine whether the re-emergence of disease in some xenografts following multidrug treatment was associated with acquired resistance to any of the agents used in the VXLD2 protocol. Leukemia cells isolated from *in vivo* drug-selected (‘R' or relapse) or saline-treated (‘C' or control) xenografts were thus tested *ex vivo* against each of the four drugs individually. Previously, we have reported that the xenografts ALL-27R and ALL-31R, which were also treated with a VXLD2 regimen *in vivo*, demonstrated acquired resistance to DEX [21] (data reproduced in Figure 2A & 2B; *p* < 0.01 for both, Repeated Measures ANOVA). Consistent with these findings, we found that ALL-42R, ALL-44R and ALL-46R showed a significant decrease in DEX sensitivity compared to their respective controls (Figure 2C, 2D and 2E; *p* < 0.01 for all, Repeated Measures ANOVA); however, there was no change in sensitivity to ASP, VCR and DNR in these xenografts (Supplementary Figure S2A, B and C).

In contrast, ALL-47R, ALL-72R and the repeated ALL-46R xenograft, showed no change in sensitivity to any of the four drugs tested compared with their controls (Supplementary Figures S2D-F; ALL-73 could not be tested in this assay due to insufficient cell numbers). In summary, DEX was the only drug for which increased resistance was observed, a phenotype observed in five out of the seven xenografts we have tested in this model so far. There thus appears to be a stochastic component to the re-emergence of disease following multidrug therapy (both in terms of the frequency with which ‘relapses' are observed, and the acquisition of a resistant phenotype) which is not reflected in the apparent stability of PCR-based clonal markers (*Ig/TCR* rearrangements).

### Analysis of genes and pathways associated with acquired DEX resistance

To better understand the biological processes associated with the acquisition of DEX resistance in this xenograft model, we performed gene expression profiling of patient diagnostic specimens and their matched control and VXLD2-treated xenografts. Array data for xenografts ALL-27 and ALL-31, previously reported [21], were also normalized and re-analyzed alongside the samples from the present study, giving a total of five xenografts with a DEX-resistance phenotype (ALL-27, ALL-31, ALL-42, ALL-44, ALL-46) and one xenograft for which no resistance was observed after drug-treatment (ALL-72); as noted above, it was not possible to determine DEX sensitivity in ALL-73 due to insufficient cell numbers.

**Figure 3:**
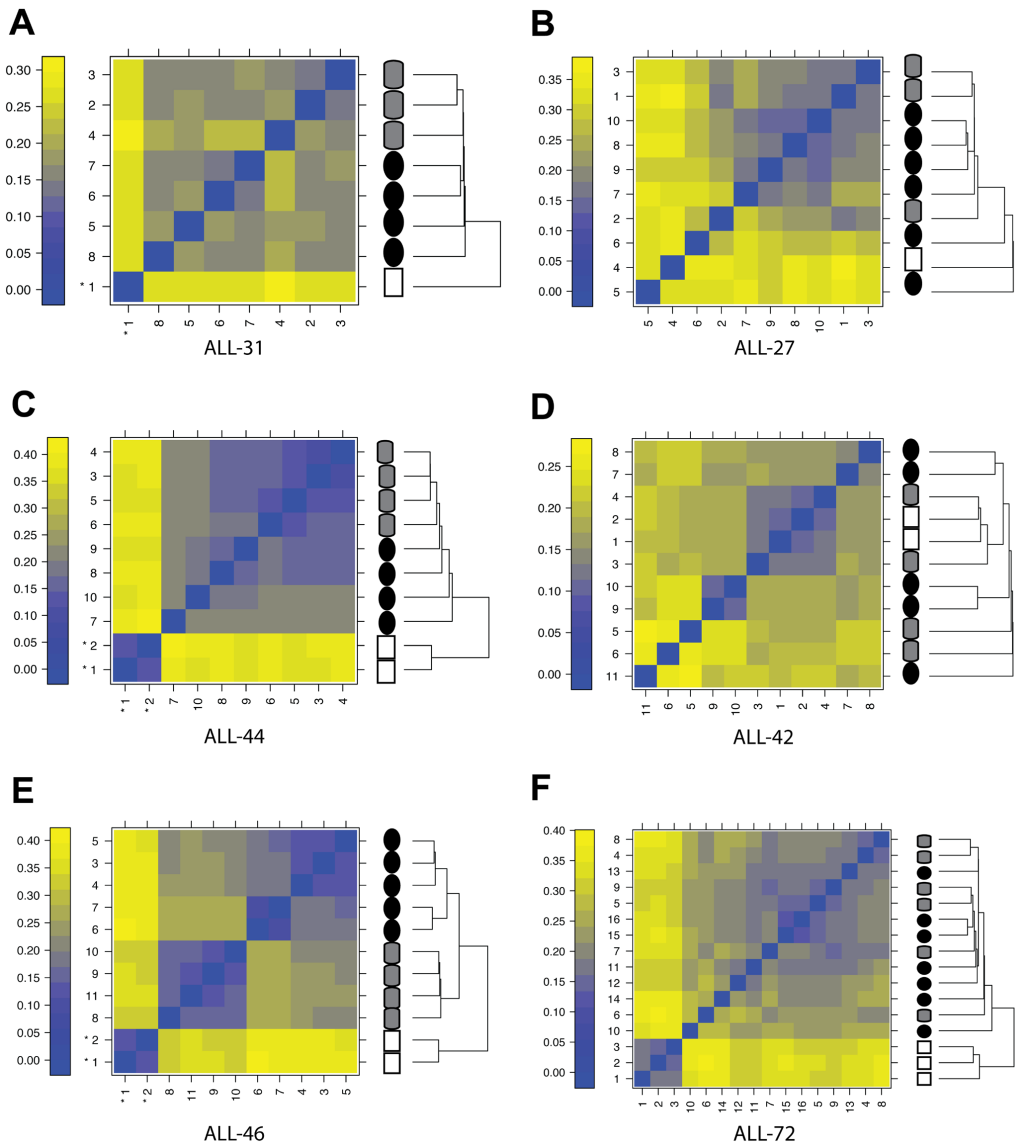
Effect of engraftment and drug-treatment on T-ALL gene expression profiles Clustering by sample similarity for patient diagnosis samples (white squares), xenograft controls (i.e. saline-treated; grey cylinders) and samples recovered following *in vivo* drug-treatment (black circles); **A.**-**E.** Xenograft lines for which VXLD2-treatment resulted in DEX resistance; **F.** Xenograft for which disease recurrence following VXDL2 was *not* associated with DEX resistance (ALL-72). Panels on the left (A, C, E) are the xenograft lines from which samples form highly distinct clusters. The color scale represents the mean absolute difference between individual arrays.

Next, we compared baseline gene-expression profiles of each of these samples using an unsupervised clustering method (Figure 3). In four out of the six xenograft lines (ALL-31, ALL-44, ALL-46 & ALL-72) the engrafted samples were clearly distinct in terms of baseline gene expression from their respective diagnostic patient specimens, and in three of these cases (Figure 3A, 3C & 3E - i.e. the left-hand panels) there were also strong transcriptional differences (distinct clusters) between control and VXLD2-treated samples. This observation was not correlated with acquired DEX resistance since neither the ALL-27 nor the ALL-42 xenografts demonstrated separation of control and VXLD2-treated samples (Figure 3B & 3D).

**Figure 4:**
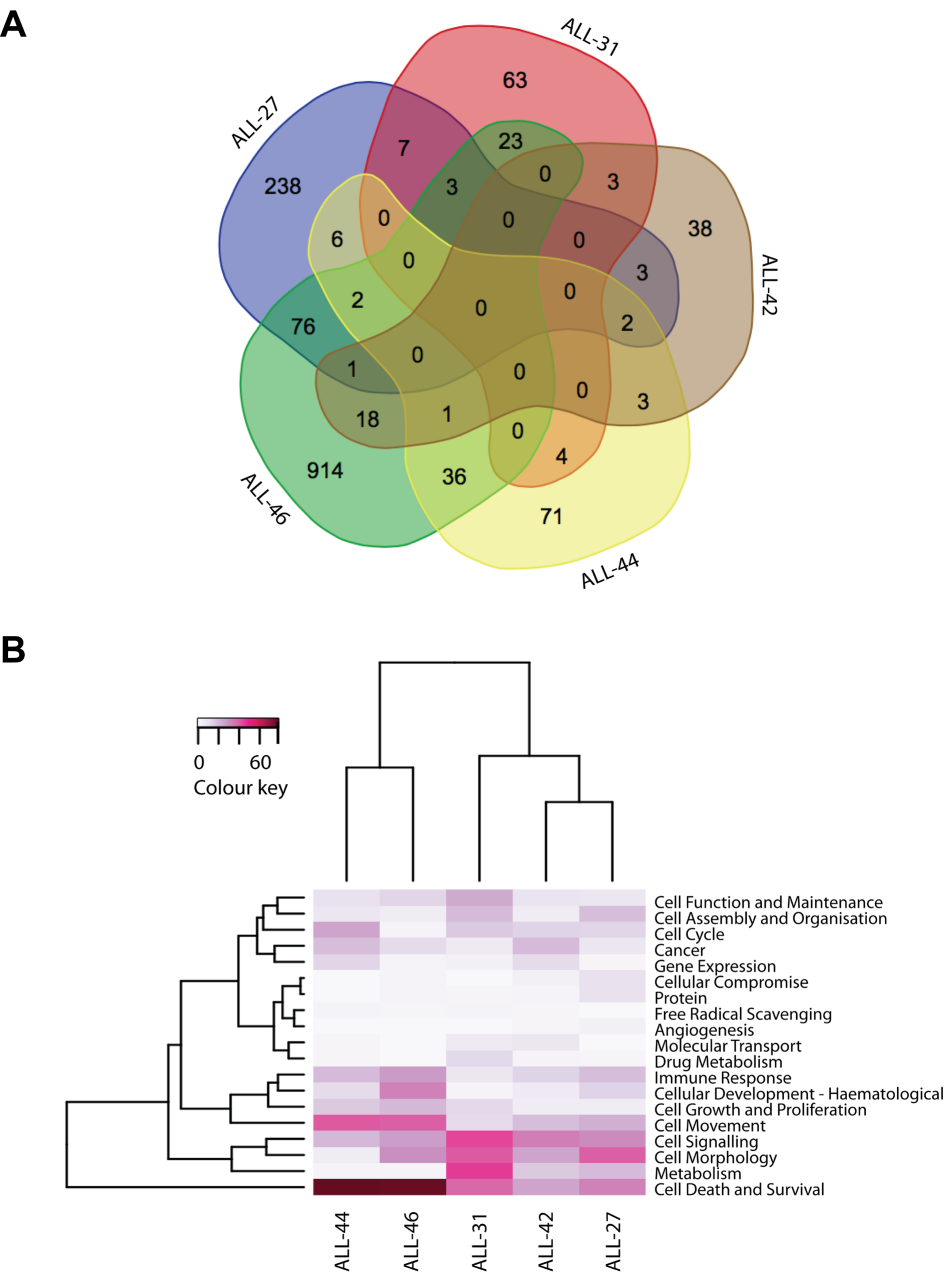
Biology of acquired DEX-resistance in VXLD2-treated xenografts **A.** Venn diagram of the lists of genes differentially expressed (adjusted *p*-value < 0.05) between control and DEX-resistant xenografts, demonstrating that the signatures associated with this acquired phenotype are largely specific to each sample; **B.** Heatmap of the most significant biological functions associated with these DEX-resistant signatures, with the color representing the absolute number (and thus the prominence) of distinct biological sub-functions associated within each category.

The clear separation of samples in ALL-31, ALL-44 and ALL-46 in Figure 3 would suggest that these xenografts should return the largest number of differentially expressed genes when comparing VXLD2 vs. control groups. However, with the exception of ALL-46 which has a particularly large number of significant genes (*n* = 1074), there was no particular pattern to the number of differentially expressed genes among the other xenografts (Figure 4A: ALL-31, *n* = 103; ALL-44, *n* = 125; ALL-27, *n* = 338; ALL-42, *n* = 69). This demonstrates that although there may be larger overall transcriptional changes in response to drug-treatment in certain xenografts (i.e. ALL-31, ALL-44 and ALL-46), a large proportion of the signal still remains specific to each biological replicate (i.e. the individual mice in each treatment arm). In other words, there is heterogeneity in the biological pathways driving DEX resistance, even within a single xenograft line.

This heterogeneity is even more evident when comparing the overlap in differentially expressed genes between the five xenografts (Figure 4A). Remarkably, there was not a single gene in common between all five analyses, demonstrating that there are multiple mechanisms by which leukemic cells may acquire resistance to DEX. A full list of the differentially expressed genes associated with each xenograft is provided in Supplementary Data File2.

To further interrogate the potential mechanism of DEX resistance, we compared the individual Affymetrix gene lists for differential regulation of ATPase Binding Cassette proteins (ABCs) and epigenetic modifiers, both of which are known to contribute to drug resistance [23, 24]. Our microarray analysis showed that ABCA2 is overexpressed in ALL-31 and ALL-46, highlighting the possible involvement of ABCs in drug resistance of these two PDXs. Furthermore, Affymetrix global gene analysis was compared to dbEM [25], an epigenetic modifier database. Three out of five relapsed xenografts showed dysregulation of epigenetic modifiers: ALL-27 showed up-regulation of SETD7 in relapse; ALL-42 showed down-regulation of KDM5B; and ALL-46 showed the most changes, with two epigenetic modifiers up-regulated (HDAC9, SMARCD3) and three down-regulated (KDM5B, HDAC7, FBXO10). Therefore, not all DEX resistant xenografts showed differential expression of ABCs or epigenetic regulators and only KDM5B was down-regulated in two out of the five xenografts. In contrast, each xenograft showed a diverse pattern of differentially regulated genes, thus emphasizing the underlying heterogeneity of drug resistance.

Given the significant heterogeneity at the level of individual genes, we used Ingenuity Pathway Analysis to assess whether there might instead be common themes in terms of underlying biological processes significantly associated with each individual xenograft showing DEX resistance (Supplementary Figure S6). The major pathways associated with DEX-resistance in each xenograft are summarized in Figure 4B, with a more detailed breakdown of the annotations belonging to each term provided in Supplementary Figure S3. Among these biological functions, the vast majority showed unique identifiers and included: tyrosine phosphorylation of protein for ALL-46, protein kinase cascade in ALL-44, MAPKKK cascade in ALL-42, small GTPase mediated signal transduction in ALL-31, and transmembrane receptor protein serine/threonine kinase signaling in ALL-27 (Supplementary Figure S6). Even at the level of biological pathways however, it is clear that there is significant heterogeneity in the mechanisms contributing to acquired DEX-resistance in these T-ALL xenografts. Through unsupervised clustering the most similar responses were seen in ALL-44 and ALL-46 - a relationship driven in large part by strong changes in cell death and survival signaling (Figure 4B; see also Supplementary Figures S4 and S5, which show the relationship of these genes within the full network of differentially expressed transcripts in ALL-44 and ALL-46). This single observation is the most notable finding from our pathway analysis, but is perhaps not surprising given the known links between glucocorticoid sensitivity and apoptotic signaling [26-29]. The overwhelming conclusion from these studies is that there is significant heterogeneity in the biological response in terms of RNA expression of these engrafted leukemias to multidrug therapy, both within individual samples and between xenografts, mirroring the individual nature of T-ALL relapse in patients.

## DISCUSSION

In this study we have implemented a novel *in vivo* approach to T-ALL xenograft drug selection, and were able to demonstrate the development of resistance even in primary cells engrafted at first passage. Out of the eight T-ALL PDX lines we have presented in this paper, five developed resistance to DEX upon disease re-emergence (ALL-27, ALL-31, ALL-42, ALL-44, ALL-46), whilst two showed no change in drug sensitivity (ALL-72 and ALL-47). These results, together with observation that the ‘repeated' engraftments for ALL-44 and ALL-46 yielded markedly different results from their respective original experiments (i.e. no re-emergence of disease in the ALL-44 repeat, and no development of drug-resistance in the ALL-46 repeat), demonstrate the variable nature of the leukemic response to therapy. It is interesting to note, that despite the use of multiple drugs during this induction therapy model we find, in accordance with clinical experience [30, 31], that when resistance is associated with disease re-emergence (i.e. relapse), it is the phenotype of dexamethasone resistance that is most commonly shared. Evidently it is this class of drug that exerts the biggest selection pressure during induction therapy, and thus must be presumed to be the most effective - explaining why sensitivity to glucocorticoids is such an important prognostic factor for ALL [8, 9, 32, 33].

Our clonal marker studies in primary patient samples showed that the predominant clones at diagnosis were preserved at relapse, consistent with clinical data [34]. This is also consistent with literature showing that leukemic clones at diagnosis are genetically highly related [19], and although gene re-arrangements may change during the course of treatment due to persistent activity of the VDJ recombination enzyme system in leukemic blasts [35] or through the selection of subclones with different Ig/TCR markers [14, 16, 36], it is unlikely that the evolution of such markers would be detectable within one month of remission induction therapy [22]. Where changes in Ig/TCR gene rearrangements in patients are observed, they are typically in cases of late relapse (>5 years from initial diagnosis) [22]. In the present study we observed that the dominant clonal rearrangements present at both patient diagnosis and relapse, were also present in corresponding control and drug-treated xenografts selected at primary, secondary or tertiary passages, indicating that these PDX models recapitulate the course of clinical disease in this regard. However, the fact that the majority of these xenografts acquired drug-resistance following treatment in the absence of changes in these standard MRD markers, demonstrates that additional phenotypic data are required to accurately predict the risk of relapse and the presence of minor drug resistant sub-clones.

In summary, it is evident that there are many different ways that cancer cells can escape from therapy, something highlighted by the heterogeneous biological signatures associated with the acquisition of DEX-resistance in the present study. The increasing sophistication of molecular genetic methods, such as next-generation sequencing, now presents the possibility of accurately tracking the *in vivo* selection of therapy-resistant clones in individual patients [12, 15, 37-39], an excellent example of which is the TRACERx Study (Tracking Cancer Evolution through Treatment [Rx]) which aims to document multi-region and longitudinal genetic changes in lung cancers to examine how they evolve, how treatment influences that process, and the impact of clonal heterogeneity on therapeutic and survival outcome [15]. While the current study focused on patients with predominantly high-risk and/or poor outcome, the application of similar strategies in the leukemia field is likely to be an important aspect of future improvements in clinical outcome for T-ALL.

## MATERIALS AND METHODS

### Ethics approval and patients

All experimental studies had received prior approval from the Animal Care and Ethics Committee of the University of New South Wales, and the Human Research Ethics Committees of the South Eastern Sydney Local Health District and the University of New South Wales. Samples used in this study were obtained from the pediatric patients treated on four different clinical trial protocols. The primary samples for ALL-27, ALL-31, ALL-44, ALL-46 and ALL-47 were obtained from children treated on Australia and New Zealand Children's Haematology and Oncology Group (ANZCHOG) ALL Study 8 clinical trial [40], whereas sample ALL-42 was obtained from an ANZCCSG ALL Study 7 [41] treated patient. ALL-72 was derived from a patient treated as per Children's Oncology Group (COG) protocol AALL0434, and ALL-73 from an 11-month old baby with ALL treated according to Interfant 06 [42, 43]. The patients were all diagnosed between 2003 and 2010 with T-ALL and referred for testing of minimal residual disease (MRD) at Children's Cancer Institute (CCI) with consent for research on biobanked material.

The patient characteristics and risk factors are shows in Table 1. Patient bone marrow aspirates were collected in Acid Citrate Dextrose tubes (Becton Dickinson, North Ryde, NSW, Australia) and layered onto Lymphoprep (Stemcell Technologies, Tullamarine, VIC, Australia), centrifuged and the mononuclear cell layer collected and washed as per manufacturer's instructions. Samples for DNA were set aside and the remaining sample was viably frozen in 1ml aliquots of 1×10^7^ or 2×10^7^ cells with fetal calf serum and 10% DMSO.

### Development of T-ALL xenografts and *in vivo* drug treatments

Xenograft lines were established as previously described [21, 44] from viably frozen T-ALL diagnosis biopsy samples, using the NOD/SCID/IL-2 receptor gamma^−/−^ (NOD.Cg-Prkdc*^scid^* Il2rg*^tm1Wjl^*/SzJ) ‘NSG' strain of immunocompromised mice. Engraftment and disease progression were monitored by enumerating the proportion of human *versus* mouse CD45^+^ cells in the peripheral blood (%huCD45^+^), using established flow cytometric procedures [13, 44]. When the %huCD45^+^ exceeded 50%, animals were euthanazed and tissues were harvested and cryopreserved. Continuous xenografts were established by inoculating spleen-derived cells from primary engrafted animals into secondary and tertiary recipient mice.

For *in vivo* drug treatment, groups of six NSG mice were inoculated intravenously with 2-5 × 10^6^ human leukemia cells, either directly from patient samples (i.e. at primary passage) or purified from the spleens of previously engrafted mice (i.e. at secondary passage). These alternative approaches were designed to test the selective pressure of the engraftment process on responses to induction-therapy and the evolution of clonal markers. In two cases (ALL-44 and ALL-46) we repeated the entire engraftment process, starting from the primary patient specimen, in order to examine variability in the selection and outgrowth of T-ALL clones in these PDX models (ALL-44 ‘repeat' and ALL-46 ‘repeat'). Mice were monitored weekly via tail vein bleeds for engraftment and subsequent to drug treatment [13, 44]. When the %huCD45^+^ reached a median of at least 1%, mice were randomized for the ‘induction-therapy' regimen VXLD2 (see Supplementary Table S1A) which we developed previously [21]. Xenografts in which leukemic cells re-emerged following VXLD2 treatment (i.e. “relapsing”) were given the designation ‘R' and their passage-matched controls the designation ‘C' (e.g. ALL-42C and ALL-42R).

To test the stability of clonal markers (Immunoglobulin and T-cell receptor, *Ig*/*TCR*, and genes) in VXLD2-selected lines, selection pressure in subsequent passages was maintained by treating xenografts with the ½ VXL module only (Supplementary Table S1A). Mice were excluded from the study if they developed spontaneous thymic lymphomas. The drugs used in this study were: DEX (Dexamethasone sodium phosphate; Hospira, Mulgrave, Victoria, Australia), VCR (Vincristine sulfate; Baxter, Darlinghurst, NSW, Australia), ASP (*L*-Asparaginase (Colaspase), Sanofi, Macquarie Park, New South Wales, Australia), DNR (Daunorubicin hydrochloride; Pfizer, West Ryde, NSW, Australia).

### Clonal analysis by identification of gene rearrangements

DNA was isolated from 1×10^7^ cells from patient diagnosis or relapse samples using the Nucleobond-AXG 100 kit (Machery Nagel, Düren, Germany) and the corresponding primary, secondary or tertiary xenografts using the DNeasy Blood and Tissue kit kit (Qiagen, Doncaster, VIC, Australia). Clonal markers were identified by PCR screening of over 300 *Ig*/*TCR* rearrangements in the *IGH, IGK*, *TRG, TRD, TRA,* *TRB* genes, as well as the *SIL-TAL1* fusion, using 24 single and multiplex PCR reactions followed by heteroduplex analysis and direct sequencing products [45-48]. Pairs of patient-specific primers were designed to enable real-time quantitative PCR (RQ-PCR) detection of the unique junctional regions (V-N-(D)-N-J), identified using NCBI IgBlast (http://www.ncbi.nlm.nih.gov/igblast/) and ESG-MRD-ALL guidelines (European Study Group on MRD detection in ALL). RQ-PCR was performed on a Biorad IQ-5 platform, using an appropriate hydrolysis probe and standard curves made by serial dilution of the patient's diagnostic DNA and normal peripheral blood mononuclear cell controls [49] according to EuroMRD guidelines [50].

### Alamar blue colorimetric assay

Xenograft cells were assessed for single agent *ex vivo* drug sensitivity by Alamar blue assay; cells were retrieved from cryostorage and resuspended in QBSF-60 medium (Quality Biological, Gaithesburg, MD, USA) supplemented with 20 ng/mL Flt-3 ligand, 100 U/mL penicillin, 100 μg/mL streptomycin, and 2 mmol/L L-glutamine. Before treatment, cells were plated in 96-well plates (100 μL/well) at a density previously optimized and were equilibrated overnight at 37°C with 5% CO_2_. Cells were then treated with 10-fold serial dilutions of each drug (DEX, VCR, ASP or DNR: 100 μM- 1nM) for 48 hours, at which point Alamar blue reagent was added (0.6 mmol/L Resazurin, 0.07 mmol/L Methylene Blue, 1 mmol/L potassium hexacyanoferrate (III), 1 mmol/L potassium hexacyanoferrate (II) trihydrate). Fluorescence was measured at 0 and 6 hours using a fluorescent plate reader (VICTOR, PerkinElmer, Glen Waverley, VIC, Australia) with excitation at 560 nm and emission at 590 nm and data are expressed as a percentage of untreated controls.

### Gene expression analysis

RNA was extracted from patient and xenograft cells using the AllPrep kit (Qiagen, Doncaster, VIC, Australia) following the manufacturer's instructions. Samples were quantified by Nanodrop ND-1000 Spectrophotometry (ThermoFisher Scientific, Scoresby, VIC, Australia) and RNA integrity assessed using the Agilent 2100 Bioanalyzer and the RNA Nano 6000 kit (Agilent Technologies, Mulgrave, VIC). Mononuclear cells were purified from xenografted spleens containing >95% huCD45^+^ cells. We have previously shown that at high engraftment levels (>90% huCD45^+^ cells) species-specific transcriptional profiles can be obtained from xenografts without requiring masking of murine signatures [51]. RNA was processed and labeled according to manufacturer's instructions (GeneChip WT Sense Target Labeling Protocol, Affymetrix, Santa Clara, CA, USA). The cDNA was hybridized onto Affymetrix GeneChip Human Gene 1.0 ST or 1.1 ST arrays), and processed according to standard protocols (Ramaciotti Centre for Genomics, Sydney, NSW, Australia).

The Bioconductor ‘arrayQualityMetrics' package [52] was used to perform quality control checks on the arrays prior to and after robust multi-array average (RMA) normalization [53]. Potential batch effects were corrected using ComBat [54] available from Bioconductor. Technical replicates were averaged prior to analysis to avoid falsely optimistic p-values. Version 19 custom chip definition files (CDFs) from the Brain Array group [55] were used to allocate Entrez gene IDs (http://brainarray.mbni.med.umich.edu/Brainarray/Database/CustomCDF/19.0.0/entrezg.asp) along with a modified affy package (affy_1.44.1) compatible with these CDFs [56]. The Bioconductor limma package [57] was used to perform differential expression analysis, adjusting p-values for multiple testing by the method of Benjamini & Hochberg [58]. Results were filtered for those genes with absolute fold changes greater than 1.5 and adjusted *p*-values < 0.05. Differentially expressed genes that passed these criteria were analyzed for biological function using Ingenuity Pathway Analysis (Ingenuity^®^ Systems; http:/www.ingenuity.com), collapsing redundant annotation terms into higher functional (parent) categories and averaging the associated *p*-values. To reduce data dimensionality, annotation terms associated with only a single sample were filtered out.

Microarray data from this study can be accessed from ArrayExpress (http://www.ebi.ac.uk/arrayexpress), accession number E-MEXP-3916 (ALL-27 & ALL-31), and E-MTAB-4759 (for all other samples).

## SUPPLEMENTARY DOCUMENT FIGURES AND TABLE






