# Supplementary material for: Heterogeneity in mechanisms of emergent resistance in pediatric T-cell acute lymphoblastic leukemia
Source: Oncotarget. 2016 Aug 11;7(37):58728–42. doi: 10.18632/oncotarget.11233 (PMC5312271; doi:10.18632/oncotarget.11233)
Supplement: Supplementary file 1 [file oncotarget-07-58728-s001.pdf]

Heterogeneity in Mechanisms of Emergent Resistance in Pediatric T-Cell Acute Lymphoblastic Leukemia

Formatted: Font color: Red

Babasaheb D Yadav[1]*, Amy L Samuels[2]*, Julia E Wells[2]*, Rosemary Sutton[3], Nicola C Venn[3], Katerina Bendak[1], Denise Anderson[4], Glenn M Marshall[5], Catherine H Cole[6], Alex H Beesley[2#], Ursula R Kees[2#] and Richard B Lock[1#].

[1]Leukaemia Biology Program, Children's Cancer Institute, Lowy Cancer Research Centre, University of New South Wales, Sydney, New South Wales, Australia; [2]Division of Children’s Leukaemia and Cancer Research, Telethon Kids Institute, University of Western Australia, Perth, Western Australia, Australia; [3]Molecular Diagnostics, Children's Cancer Institute, Lowy Cancer Research Centre, University of New South Wales, Sydney, New South Wales, Australia [4]Division of Bioinformatics and Biostatistics, Telethon Kids Institute, University of Western Australia, Perth, Western Australia, Australia; [5]Kids Cancer Centre, Sydney Children’s Hospital, Sydney, New South Wales, Australia [6]School of Paediatrics and Child Health, University of Western Australia, Perth, Western Australia, Australia

* Denotes Equal First Authorship

# Denotes Equal Senior Authorship

SUPPLEMENTARY DOCUMENT

Supplementary Figure S1. Repeat experiments to test reproducibility of engraftment kinetics for (A) ALL-46 and (B) ALL-44. NSG mice were inoculated with the respective patient diagnosis sample and treated with VXLD2 or saline in an identical fashion to the initial experiments. The percentage of human CD45$^+$ cells in the peripheral blood was used to assess leukaemia progression.

A **ALL-46 repeat**

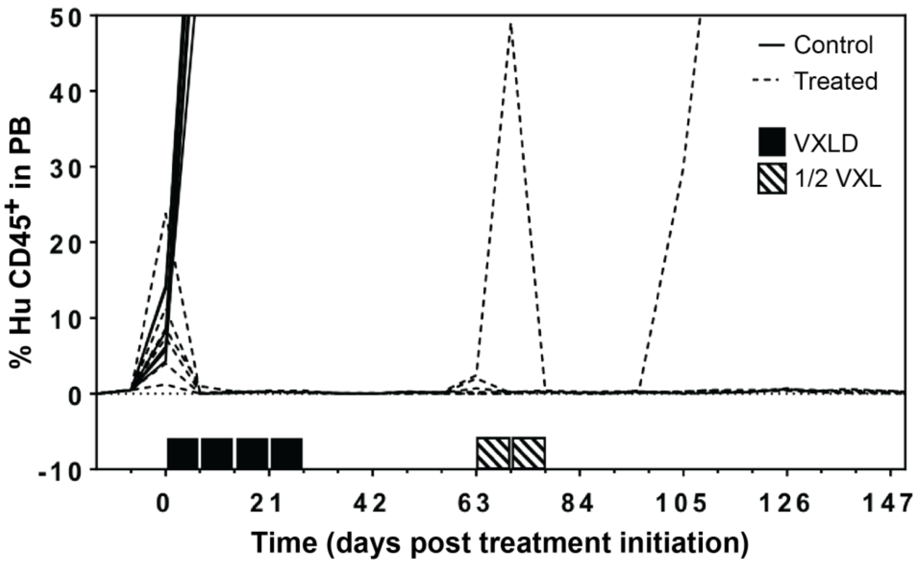

B **ALL-44 repeat**

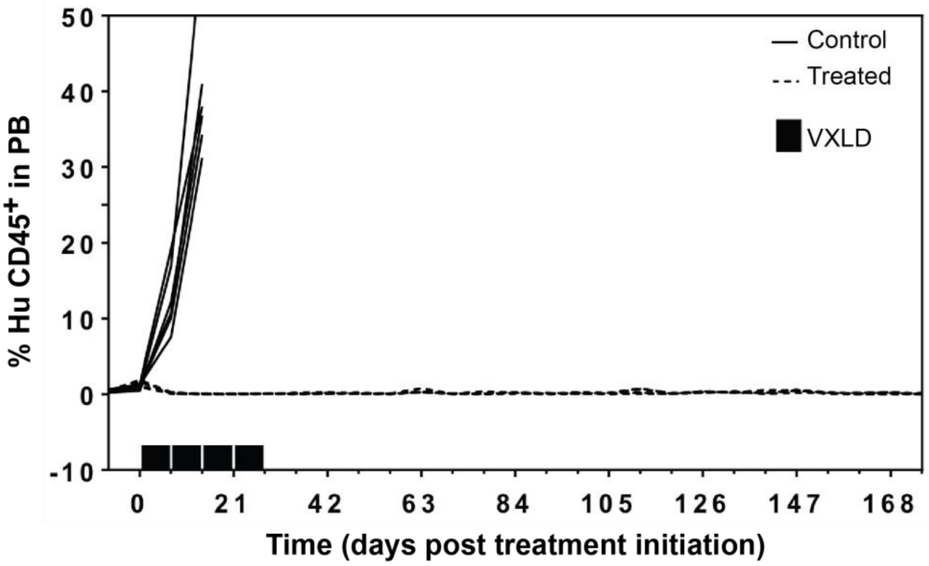

Supplementary Figure S2. *Ex vivo* assessment of drug resistance in xenografts with re-emergence of disease following VXLD2 ‘induction therapy’. Saline treated control samples (‘C’) or their treated counterparts (‘R’ or relapse) from each xenograft line (A, ALL-42; B, ALL-44; C, ALL-46; D, ALL-46 ‘repeat’ experiment; E, ALL-47; F, ALL-72) were assessed for altered sensitivity to single agent L-asparaginase, vincristine or daunorubicin (A-F) or dexamethasone (D-F), by Alamar blue assay. Three replicate experiments were performed in each xenograft subline and the median survival is depicted.

A

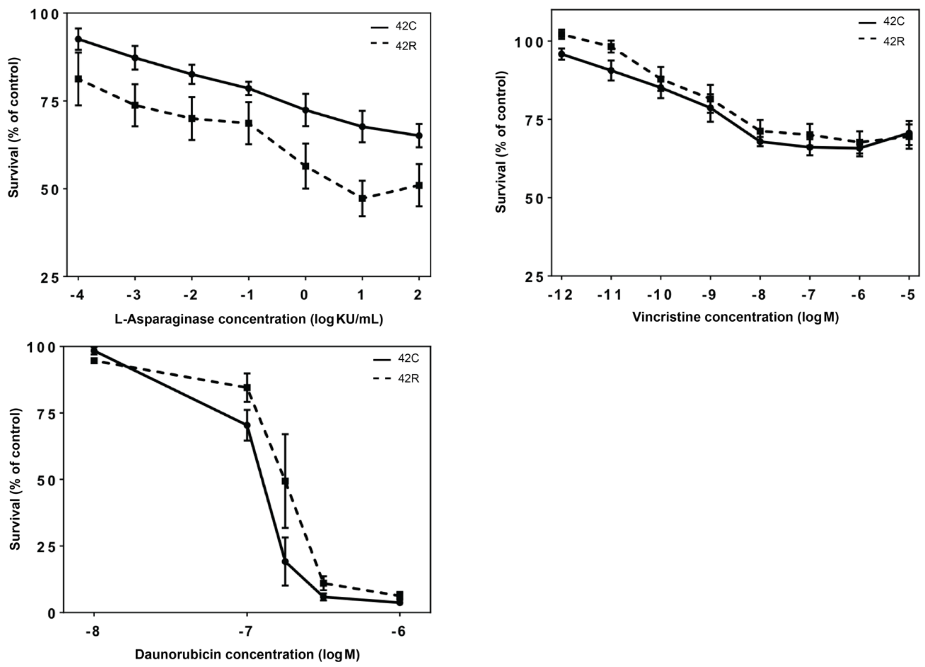

B

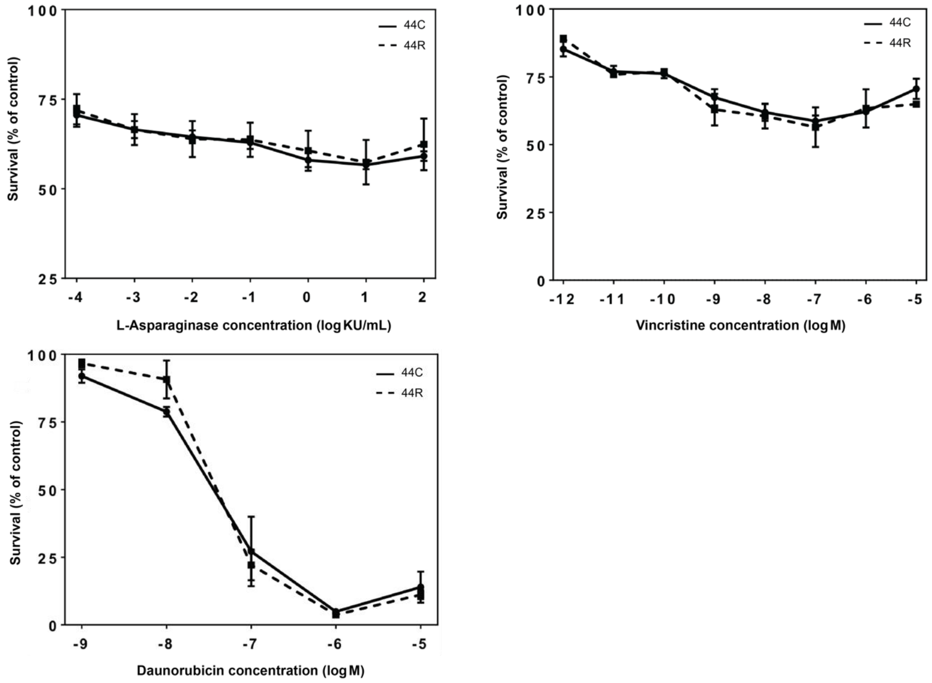

C

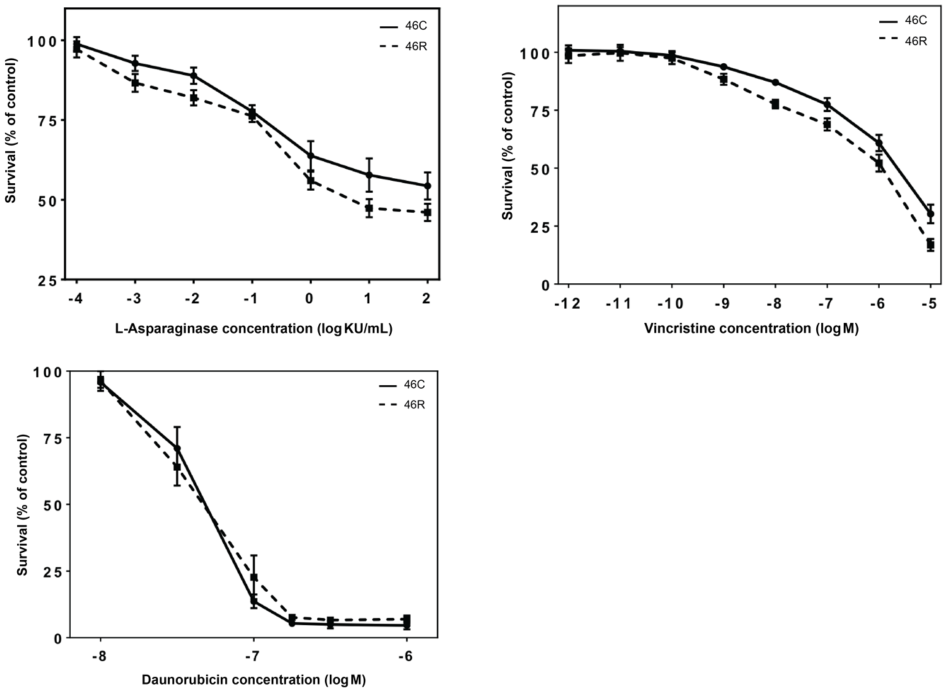
46C
46R
Survival (% of control)
L-Asparaginase concentration (log KU/mL)
Vincristine concentration (log M)
Daunorubicin concentration (log M)

D

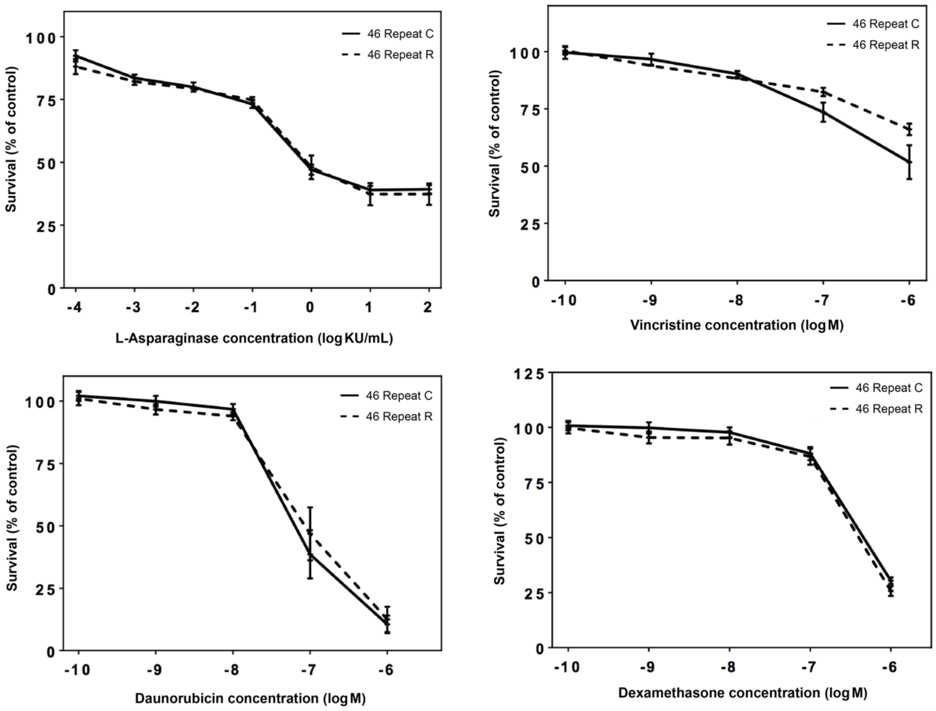
46 Repeat C
46 Repeat R
Survival (% of control)
L-Asparaginase concentration (log KU/mL)
Vincristine concentration (log M)
Daunorubicin concentration (log M)
Dexamethasone concentration (log M)

E

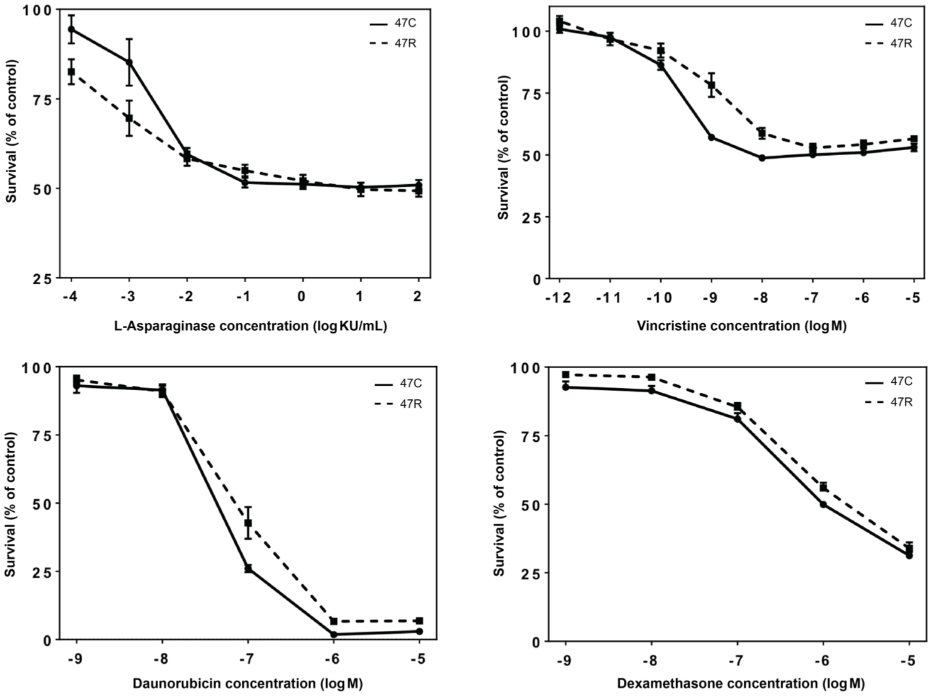
47C
47R
Survival (% of control)
L-Asparaginase concentration (log KU/mL)
Vincristine concentration (log M)
Daunorubicin concentration (log M)
Dexamethasone concentration (log M)

F

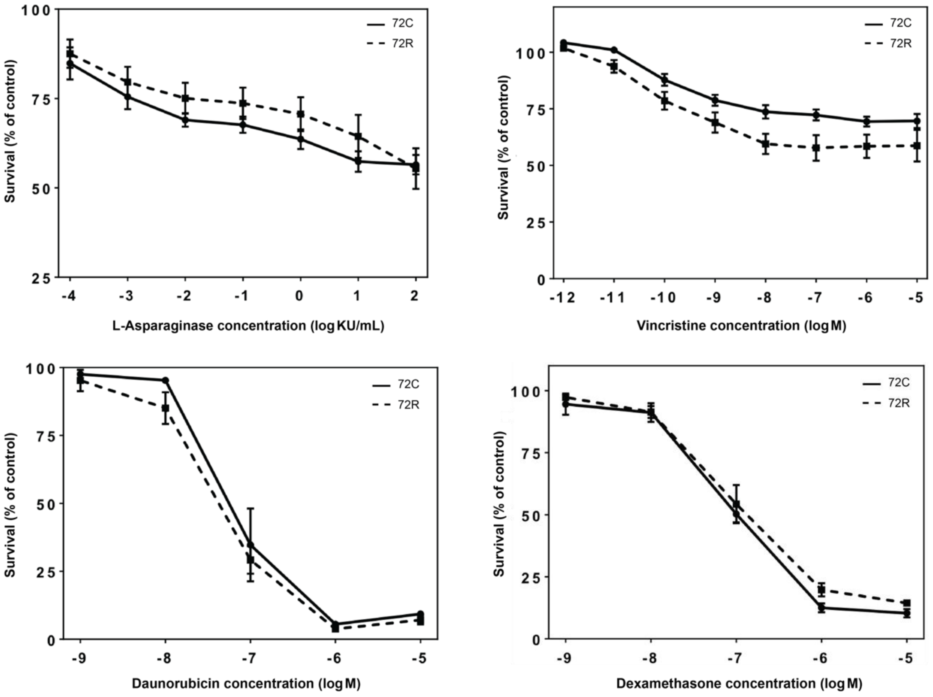
72C
72R
Survival (% of control)
L-Asparaginase concentration (log KU/mL)
Vincristine concentration (log M)
Daunorubicin concentration (log M)
Dexamethasone concentration (log M)

Supplementary Figure S3. Biological functions of genes differentially expressed between control and VXLD2 samples in at least two xenografts (Ingenuity Pathway Analysis). Parent (higher) biological function terms are indicated on the left. The colour key represents negative log p-values (Fisher Exact Test) of the child annotations on the right.

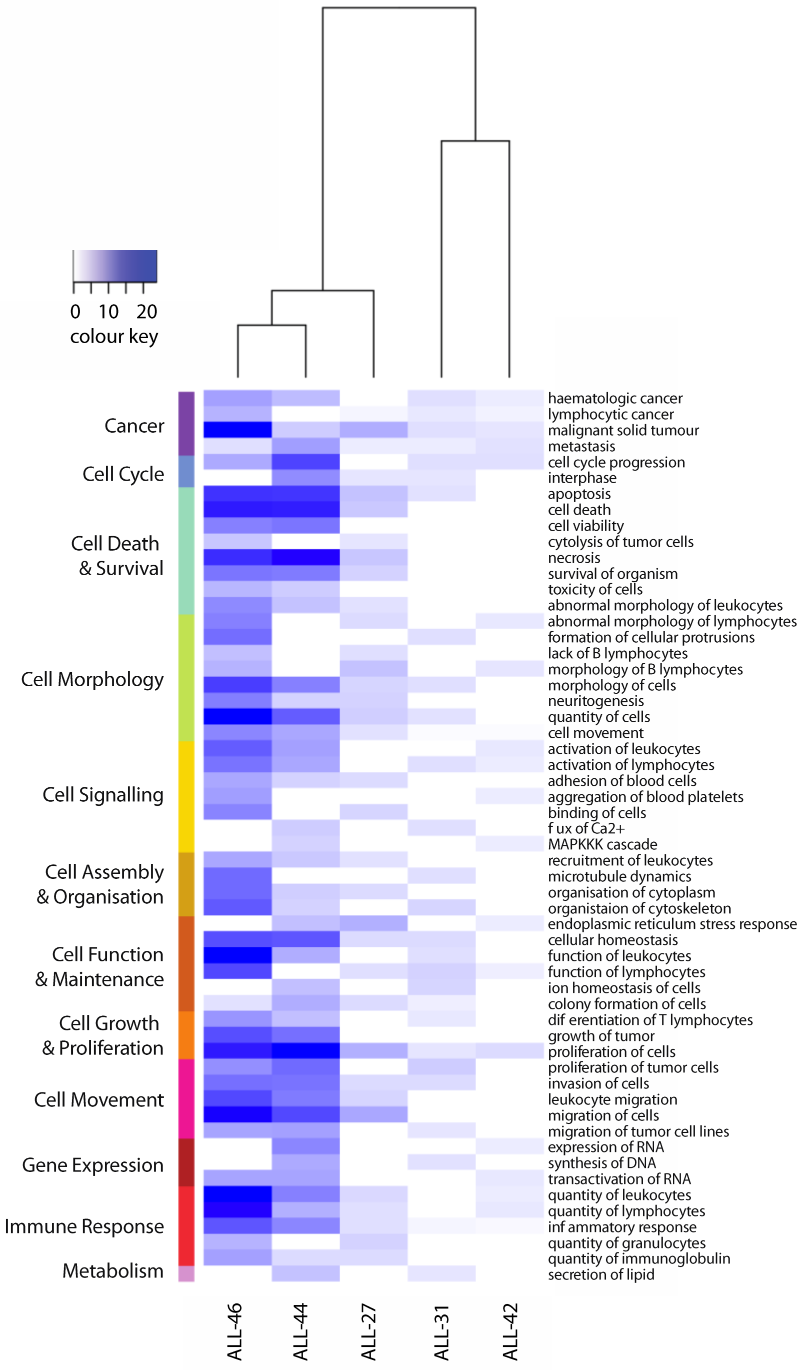

Supplementary Figure S4. Network analysis of genes associated with the acquisition of DEX-resistance in ALL-44, highlighting those members (red hubs) with gene ontology terms for cell death and survival (STRING database; http://string-db.org/).

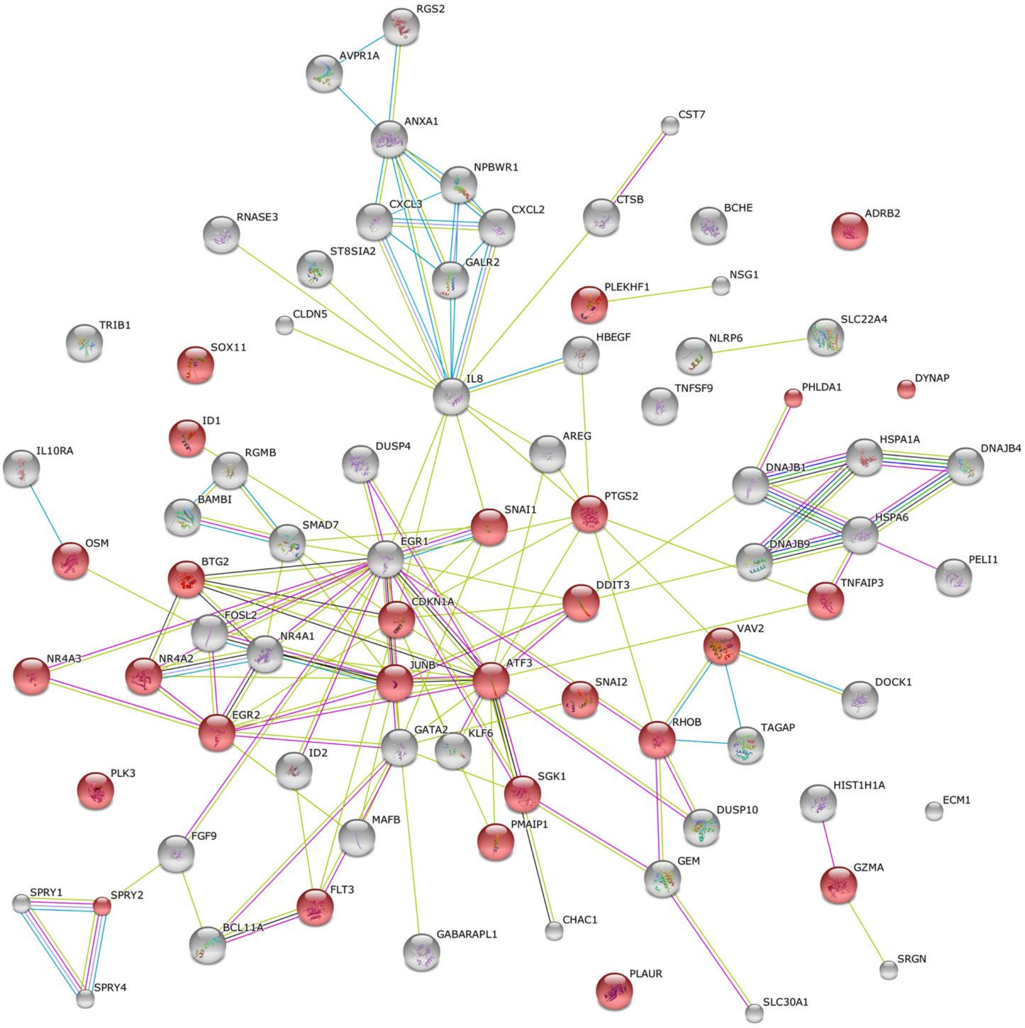

Supplementary Figure S5. Network analysis of genes associated with the acquisition of DEX-resistance in ALL-46, highlighting those members (red hubs) with gene ontology terms for cell death and survival (STRING database; http://string-db.org/); for visualisation purposes, the outer edges of network have been trimmed.

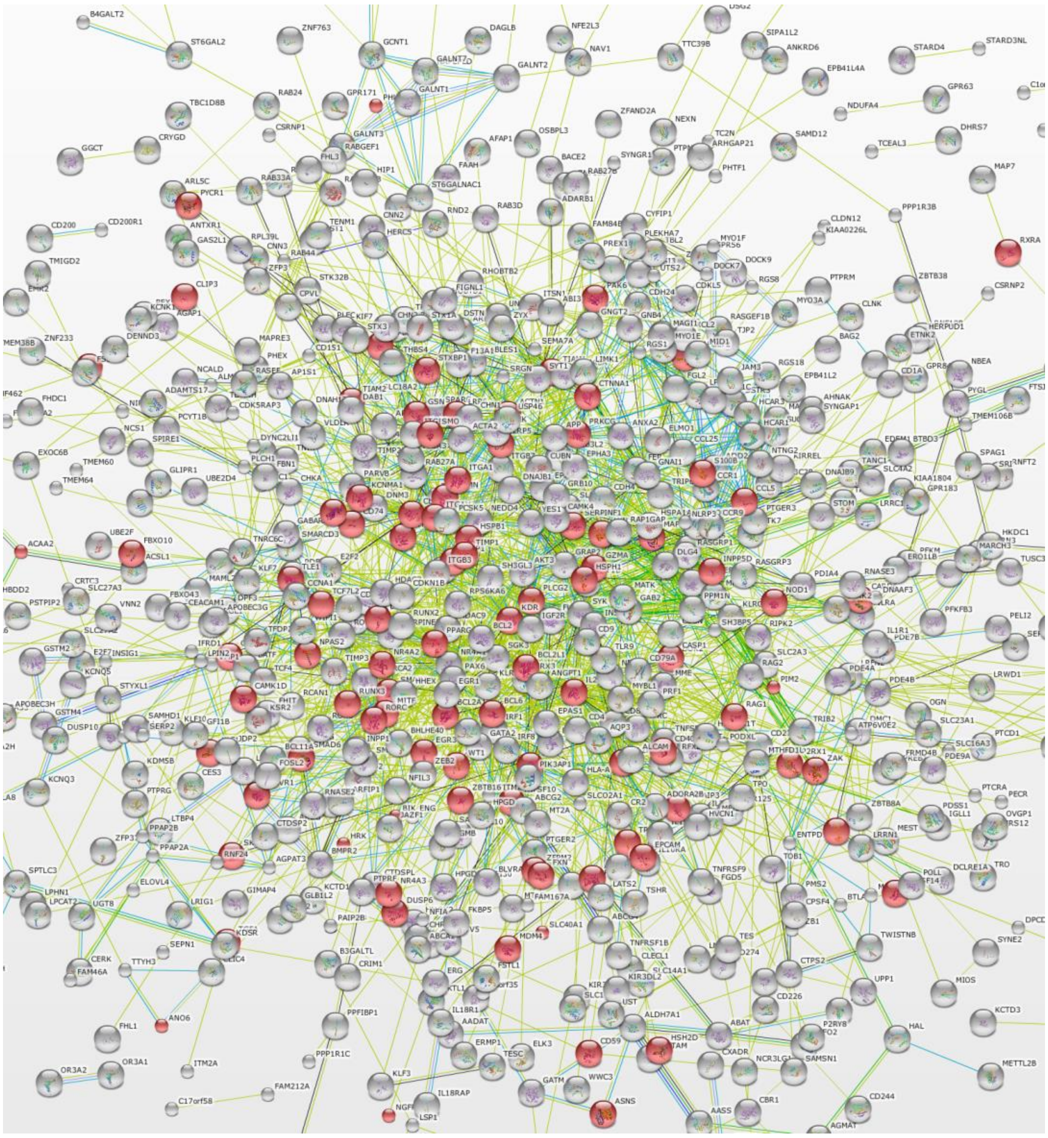

Supplementary Figure S6. Top ranked biological functions associated with differentially expressed genes between control and VXLD2 samples for each DEX-resistant xenograft (Ingenuity Pathway Analysis). (A) ALL-46, (B) ALL-44, (C) ALL-42, (D) ALL-31 and (E) ALL-27. Biological functions were ranked by negative log p-values (Fisher Exact Test) and filtered for non-relevant and redundant functions.

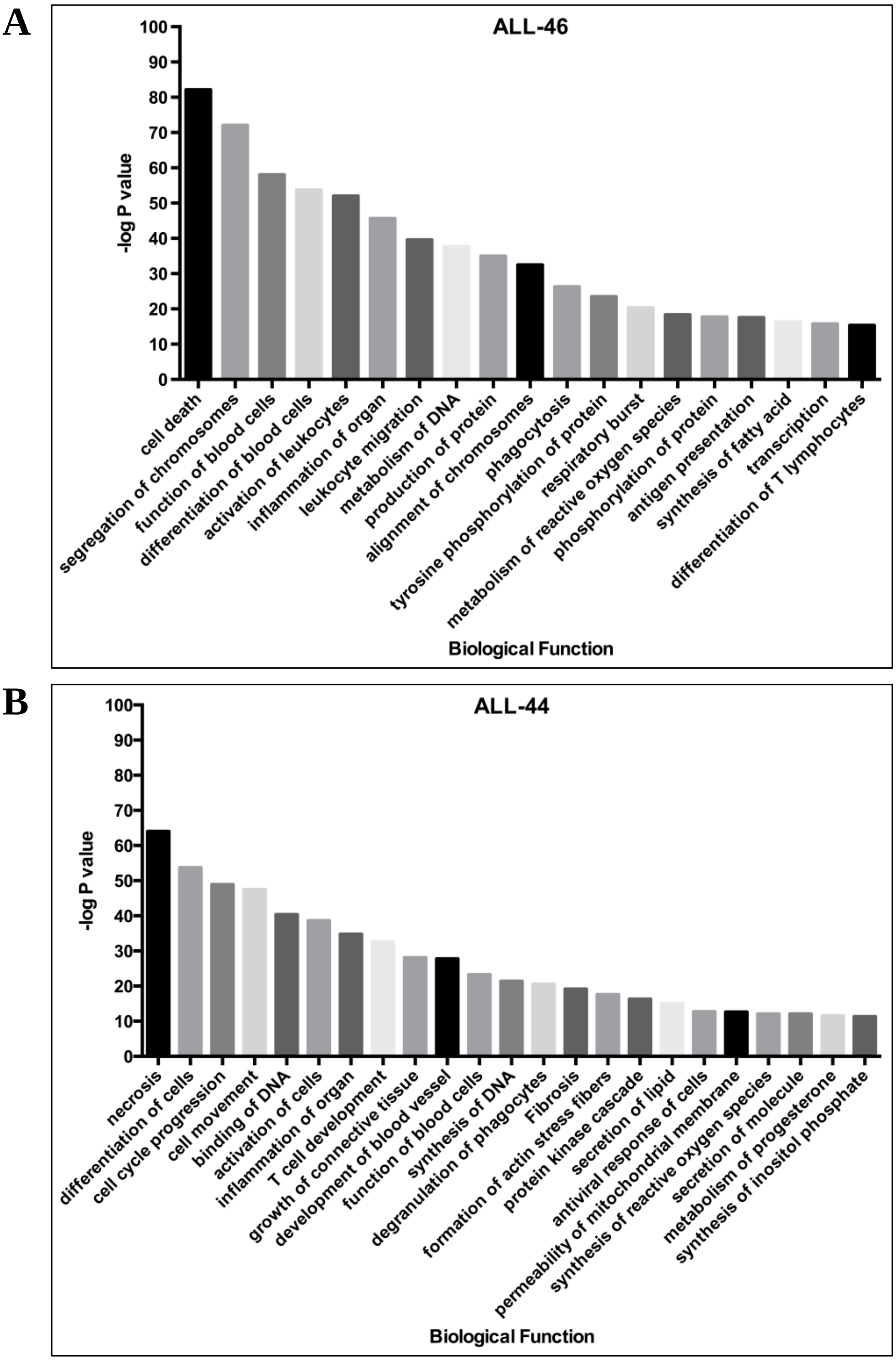

C

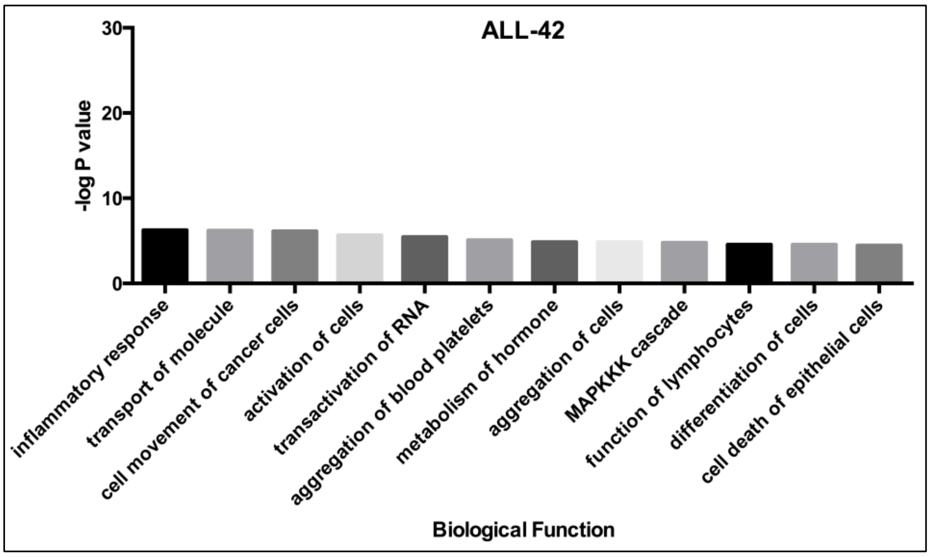
ALL-42
-log P value
30
20
10
0
inflammatory response
transport of molecule
cell movement of cancer cells
activation of cells
transactivation of RNA
aggregation of blood platelets
metabolism of hormone
aggregation of cells
MAPKKK cascade
function of lymphocytes
differentiation of cells
cell death of epithelial cells
Biological Function

D

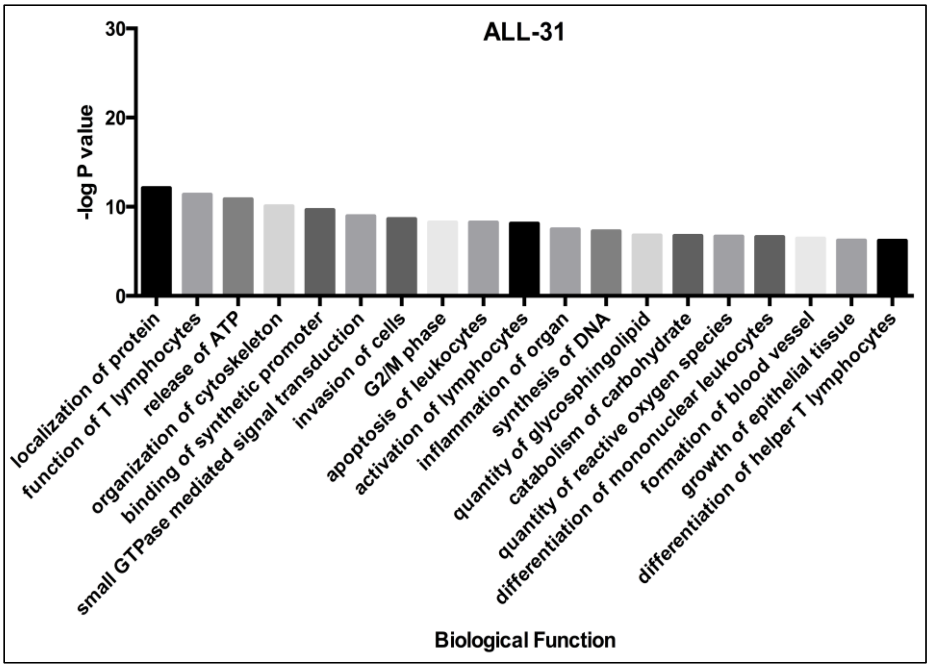
ALL-31
-log P value
30
20
10
0
localization of protein
function of T lymphocytes
release of ATP
organization of cytoskeleton
binding of synthetic promoter
small GTPase mediated signal transduction
invasion of cells
G2/M phase
apoptosis of leukocytes
activation of lymphocytes
inflammation of organ
synthesis of DNA
quantity of glycosphingolipid
catabolism of carbohydrate
quantity of reactive oxygen species
differentiation of mononuclear leukocytes
formation of blood vessel
growth of epithelial tissue
differentiation of helper T lymphocytes
Biological Function

E

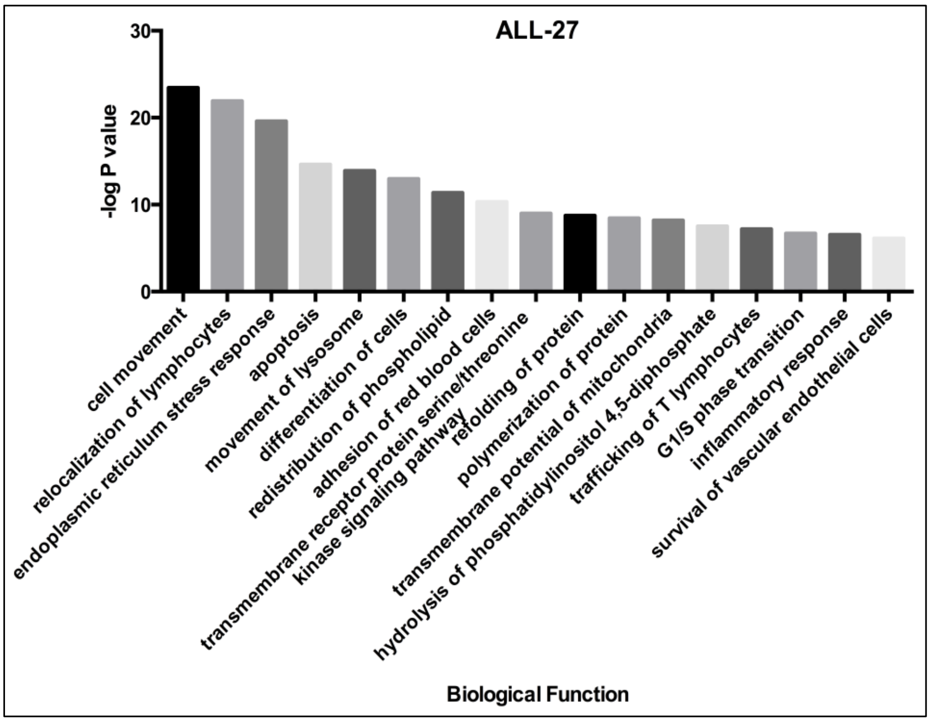
ALL-27
30
20
10
0
-log P value
cell movement
relocalization of lymphocytes
endoplasmic reticulum stress response
apoptosis
movement of lysosome
differentiation of cells
redistribution of phospholipid
adhesion of red blood cells
transmembrane receptor protein serine/threonine kinase signaling pathway
refolding of protein
polymerization of protein
transmembrane potential of mitochondria
hydrolysis of phosphatidylinositol 4,5-diphosphate
trafficking of T lymphocytes
G1/S phase transition
inflammatory response
survival of vascular endothelial cells
Biological Function

Supplementary Table S1. Chemotherapeutic treatment regimen of xenografts. (**A**) Chemotherapeutics used in each treatment module; (**B**) Design of the "VXLD2" treatment regimen used for the generation of multidrug resistant patient-derived xenografts.

A

Treatment Module	Drugs Used	Concentration	Treatment schedule
VXLD<br>(induction therapy combining all 4 drugs)	Vincristine ("V")<br>Dexamethasone ("X")<br>L-asparaginase ("L")<br>Daunorubicin ("D")	0.15 mg/kg/day i.p.<br>5 mg/kg/day i.p.<br>1000 kU/kg/day i.p.<br>2.5 mg/kg/day i.v.	1 x week (4 weeks)<br>Mon – Fri (4 weeks)<br>Mon – Fri (4 weeks)<br>1 x week (4 weeks)
½ VXL<br>(re-induction therapy with 3 drugs at half-dose i.e. no daunorubicin)	Vincristine ("V")<br>Dexamethasone ("X")<br>L-asparaginase ("L")	0.075 mg/kg/day i.p.<br>2.5 mg/kg/day i.p.<br>500 kU/kg/day i.p.	1 x week (2 weeks)<br>Mon – Fri (2 weeks)<br>Mon – Fri (2 weeks)

B

Xenograft	Treatment schedule "VXLD2"				Number of evaluable samples recovered/mice inoculated
	Block 1		Block 2		
	VXLD treatment	No treatment	½ VXL treatment	No treatment	
ALL-42	4 wks	Until relapse	2 wks	Until relapse	5/6
ALL-44	4 wks	Until relapse	2 wks	Until relapse	4/6
ALL-44 (Repeat)	4 wks	Until relapse	2 wks	Until relapse	0/6
ALL-46	4 wks	Until relapse	2 wks	Until relapse	1/6
ALL-46 (Repeat)	4 wks	Until relapse	2 wks	Until relapse	1/6
ALL-47	4 wks	Until relapse	2 wks	Until relapse	5/6
ALL-72	4 wks	Until relapse	2 wks	Until relapse	6/6
ALL-73	4 wks	Until relapse	2 wks	Until relapse	4/6
